# Intra-Articular Injection of 2 Different Dosages of Autologous and Allogeneic Bone Marrow- and Umbilical Cord-Derived Mesenchymal Stem Cells Triggers a Variable Inflammatory Response of the Fetlock Joint on 12 Sound Experimental Horses

**DOI:** 10.1155/2019/9431894

**Published:** 2019-05-02

**Authors:** Lélia Bertoni, Thomas Branly, Sandrine Jacquet, Mélanie Desancé, Loïc Desquilbet, Pascaline Rivory, Daniel-Jean Hartmann, Jean-Marie Denoix, Fabrice Audigié, Philippe Galéra, Magali Demoor

**Affiliations:** ^1^CIRALE, USC 957, BPLC, INRA, Ecole Nationale Vétérinaire d'Alfort, Maisons-Alfort F-94700, France; ^2^Normandie University, UNICAEN, BIOTARGEN, 14000 Caen, France; ^3^U955-IMRB, Inserm, Ecole Nationale Vétérinaire d'Alfort, UPEC, Maisons-Alfort F-94700, France; ^4^NOVOTEC, ZAC du Chêne, Europarc, 69500 Bron, France

## Abstract

Osteoarthritis is a significant and costly cause of pain for both humans and horses. The horse has been identified as a suitable model for human osteoarthritis. Regenerative therapy with allogeneic mesenchymal stem cells (MSCs) is a promising treatment, but the safety of this procedure continues to be debated. The aim of this study is to evaluate the safety of intra-articular injections of allogeneic MSCs on healthy joints by comparing two different dosages and two different tissue sources, namely, bone marrow and umbilical cord blood, with a placebo treatment on the same individuals. We also assessed the influence of autologous versus allogeneic cells for bone marrow-derived MSC treatment. Twelve clinically sound horses were subjected to injections in their 4 fetlock joints. Each of the three fetlocks was administered a different MSC type, and the remaining fetlock was injected with phosphate-buffered saline as a control. Six horses received 10 million cells per joint, and the 6 other horses received 20 million cells per joint. Clinical and ultrasound monitoring revealed that allogeneic bone marrow-derived MSCs induced significantly more synovial effusion compared to umbilical cord blood-derived MSCs but no significant difference was noted within the synovial fluid parameters. The administration of 10 million cells in horses triggered significantly more inflammatory signs than the administration of 20 million cells. Mesenchymal stem cell injections induced mild to moderate local inflammatory signs compared to the placebo, with individual variability in the sensitivity to the same line of MSCs. Understanding the behavior of stem cells when injected alone is a step towards the safer use of new strategies in stem cell therapy, where the use of either MSC secretome or MSCs combined with biomaterials could enhance their viability and metabolic activity.

## 1. Introduction

Osteoarthritis (OA) is a joint disease characterized by cartilage breakdown, subchondral bone failure, periarticular bone remodeling, and synovitis [1–3]. It is one of the leading causes of lameness, reduced performance, and early retirement in athletic horses [4]. This disease is directly and indirectly responsible for major financial loss in the equine industry, making the development of curative therapeutic strategies a subject of great interest. This interest is particularly enhanced as the horse is a suitable model to study human OA. Osteoarthritis is indeed responsible for a great number of welfare and economic concerns in humans [3]. Although many medical approaches [3] have been evaluated or used, the effects of these treatments remain palliative (pain reduction, decreased inflammation) and there is currently no curative treatment for OA. The same concerns are also applicable for recent surgical approaches that appear promising and are still under consideration [5].

Stem cell-based regenerative medicine is a promising strategy given the lack of spontaneous regenerative capacity in the articular cartilage. The expected effects are explained by the chondrogenic differentiation potential of stem cells and their immunomodulatory and paracrine signaling effects [6, 7]. Allogeneic MSCs are particularly interesting because banking can be performed. This provides characterized cells of known quality and quantity that are readily available for immediate treatment, whereas autologous MSCs require time to be isolated and expanded in culture with uncertain results about the quality of the cells obtained. Despite the fact that relatively little is known about their *in vivo* biology, intra-articular injections of MSCs have already been carried out in the clinic [8], and a number of commercial laboratories throughout the world can now process various tissues to generate stem cells for clients. However, the occurrence of adverse reactions after intra-articular injections of MSCs has already been reported in equine patients [8, 9], and it is not uncommon in clinical practice for nonsteroidal anti-inflammatory drugs (NSAIDs) to be administrated prior to MSC injection to reduce the risk of joint flare, as recommended [9, 10]. It is thus important not to overestimate the safety of MSC treatment, and many questions need to be addressed before generalizing the use of these cells in clinical practice. *In vitro* [11] and *in vivo* studies [12–14] show that MSCs are indeed commonly thought to be immunoprivileged, but there is controversy about the local inflammatory responses observed following *in vivo* intra-articular injection [15–17] and the potential immunogenicity of these cells [18–20]. Although some recent studies have evaluated the safety of allogeneic equine MSCs [21], only a small number were performed using an intra-articular approach: three focused on BM-MSCs [15, 17, 20], one focused on placenta-derived MSCs [16], and one described chondrogenically induced MSCs derived from peripheral blood combined with allogeneic plasma [22]. In these study designs, each horse received just one type of MSC, and a single dosage of MSCs was tested, with doses ranging from 2 to 25 million cells per joint depending on the studies. The situation is the same for human studies: a recent review [23] reveals that there is currently no consensus on the best type or dose of MSCs for injection into joints to ensure for a safe and potentially effective treatment of OA lesions. To our knowledge, no study to date compares on the same individuals the safety of MSCs from two different tissue sources on healthy joints or compares the use of different dosages using the same protocol.

The objectives of this study are twofold. Firstly, we sought to compare the safety of intra-articular injections in the healthy joints of 2 different allogeneic MSC tissue sources using cells of controlled quality and viability and consisting of BM-MSCs and UCB-MSCs with phosphate-buffered saline (PBS) as the vehicle control. The influence of autologous versus allogeneic cells was also assessed for BM-MSCs. Secondly, we compared the safety of these intra-articular injections with 10 million cells versus 20 million cells.

## 2. Materials and Methods

### 2.1. Horses and Study Design

Twelve clinically sound French Standardbreds owned by the Center of Imaging and Research on Equine Locomotor Pathology (CIRALE) were included in the study. There were 7 geldings and 5 mares, all aged from 2 to 4 years old. None of the horses had a history of pregnancy, had received a blood transfusion before recruitment, or had kin relationships. Each horse was evaluated clinically with static and dynamic evaluation and underwent radiographic and ultrasonographic examination of its four fetlock joints to rule out the presence of preexisting signs of articular disease. The study protocol was approved by the ComEth Anses/ENVA/UPEC Ethical Committee (permit number: 10/06/14-8). All horses received a single injection in all 4 fetlock joints, i.e., in both metacarpophalangeal and both metatarsophalangeal joints. A uniform distribution of the different stem cell types was performed in which each horse had one front/hind fetlock joint injected with autologous BM-MSCs and its contralateral fetlock joint injected with allogeneic BM-MSCs and one front/hind fetlock joint injected with UCB-MSCs and its contralateral fetlock joint injected with the same volume of the MSC transport medium (Gibco Phosphate-Buffered Saline, Fisher Scientific SAS, Illkirch, France) as a control. Each horse was randomly assigned to one of the six stem cell treatment distributions (see Supplementary 7). Injections were performed by the same trained operator who was not informed about the choice of stem cell distributions (SJ, ACVSMR diplomate). Individuals were sedated prior to the injection (IV administration of a combination of detomidine 0.01 mg/kg and butorphanol 0.01 mg/kg) after aseptic preparation of the skin. As recommended to ensure MSC viability [24], injections were performed using a lateral approach on the flexed limb with a 20-gauge needle inserted between the metacarpal/metatarsal condyle and the lateral proximal sesamoid bone (Supplementary 4). A uniform distribution of BM-MSCs and UCB-MSCs was performed for the left and right fetlocks and the front and hind fetlocks. The horses were divided into 2 groups. The horses in group 1 received 2 mL containing 20 million MSCs in transport medium, whereas the horses in group 2 received 2 mL containing 10 million MSCs in transport medium. PBS was used as a transport medium. A pilot study on two healthy horses had previously confirmed the good tolerance of intra-articular PBS injections in view of the absence of synovial effusion and sensitivity to flexion tests of the joints injected, as well as the absence of lameness during a two-week clinical follow-up (the protocol used was identical to the one used in this study).

### 2.2. Cell Isolation and Cell Culture

Sternal bone marrow was taken from each of the 12 horses after sedation (detomidine 0.01 mg/kg, IV; butorphanol 0.02 mg/kg, IV) and local skin anesthesia, as previously described **[** 25–27 **]**. Briefly, 30 mL of bone marrow was drawn by suction using an 11G Jamshidi biopsy needle in syringes preloaded with heparin. The bone marrow samples were collected into sterile flasks containing 40 mL of citrate phosphate dextrose anticoagulant then stored at room temperature and processed within 1 to 2 hours of collection. The blood from the equine umbilical cord blood was collected from twenty-four foals immediately after foaling by venipuncture of the umbilical vein using a 16G hypodermic needle attached to a 250 mL blood transfusion collection bag (MSE3500Q, Macopharma) containing 35 mL of citrate phosphate-dextrose-adenine, as previously described [28, 29]. Umbilical cord blood samples were then stored at room temperature (19-22°C) and processed within 9 to 62 hours of collection.

MSC isolation and culture were performed in the same manner and using the same culture medium as previously described in *in vitro* studies [26, 27, 29]. To ensure the safety of isolated cells, bacteriological and virological analyses were carried out targeting nine viral genera, eight bacterial genera, and two protozoa (see Supplementary 7) by an external laboratory according to their internal protocols (Labéo Frank Duncombe, Saint-Contest, France). Positive samples were only found for Herpesvirus (71% for UCB-MSCs and 12.5% for BM-MSCs) [26, 29]. Positive cell lines were excluded from further allogeneic administration.

Cell expansion was performed in low-glucose Dulbecco's modified Eagle medium containing 20% fetal calf serum (FCS, Invitrogen Life Technologies). The culture medium was changed three times per week and cells were passaged at 80% confluency until passage 4 (P4). At this stage, cells kept per cell lines were counted, centrifuged, and then suspended in a cryopreserved medium (6 to 10 million cells/mL) composed of 90% FCS and 10% dimethyl-sulfoxide (Sigma-Aldrich). Freezing was performed using CoolCell freezing containers and cells were stored in liquid nitrogen until needed for the study.

### 2.3. Cell Characterization In Vitro

Immunophenotyping and trilineage differentiation were performed as previously described [26, 29] on cells prepared from the same passage and in the same manner as those used for clinical injections. Briefly, MSCs at P4 were immunophenotyped for expression levels of MHC class II and for a panel of markers using flow cytometry. The following mouse monoclonal anti-human antibodies were used: CD29-Allophycocyanin (APC) (BioLegend), CD44-Phycoerythrin (PE) (IOTest), CD45-Pacific Blue (PB) (ABd Serotec), CD73-APC (Abcam), CD90-Fluorescein isothiocyanate (FITC) (InvestCare), CD105-FITC (Abcam), and type II MHC-RPE (ABd Serotec). The respective mouse isotype antibodies were used as controls. These monoclonal antibodies crossed with the horse cells, and specificity was verified by running a blood sample, containing mononuclear cells, in the cytometer as a positive control. Data acquisition was performed in a Beckman Coulter Gallios flow cytometer (Federative Research Structure ICORE platform, University of Caen Normandy, France) and analyzed with FlowJo Software (TreeStar).

The capacity of equine MSCs to differentiate into osteogenic, chondrogenic, and adipogenic lineages was determined at P4 using culture and fixation methods described previously [26, 29]. Briefly, osteogenic differentiation was assessed by using Alizarin Red S staining to evaluate calcium deposition, adipogenesis by using Oil Red O staining to observe lipid droplets, and chondrogenesis by using Alcian Blue staining to observe acidic polysaccharides such as glycosaminoglycans. During each differentiation assay, an equal number of cells were maintained in the MSC expansion medium as a control and stained for analysis.

### 2.4. Cell Preparation for Injection

Allogeneic sources of cells were randomly selected from available cell lines with 2 lines of BM-MSCs per group of horses (group 1: dosage 20 million; group 2: dosage 10 million) and 2 lines of UCB-MSCs per group. Approximately one week before the intra-articular injections (7 days for BM-MSCs and 10 days for UCB-MSCs), cells were thawed rapidly by friction, seeded at 5000 cells/cm^2^, and cultured with the medium described in the cell culture section. Two to three hours before injection, cells were detached using trypsin/EDTA then suspended in 50 mL of PBS to completely remove the culture medium and especially the residual fetal bovine serum (FBS). Cells were then counted before a second centrifugation. Six batches of each MSC source containing 10 million cells/mL of PBS were prepared for injection for group 1, and 6 batches containing 5 million cells/mL of PBS were prepared for injection for group 2. For allogeneic injections, cell lines were randomly assigned to recipient horses (see paring in Supplementary 2). MSCs were maintained at room temperature (19-22°C) during transport from the laboratory, as recommended [30]. A trypan blue exclusion test (Trypan Blue solution 0.4% liquid, Merck) was used to monitor cell viability for 6 hours after culture removal: a manual count was performed immediately after mixing of a fraction of the medium containing the cells into the trypan blue solution at a ratio of 1 : 1.

### 2.5. Clinical and Imaging Follow-Up

All horses were confined to 3 × 4 m stalls from the day before injections until the day after. They were then turned out in small paddocks (10 × 10 m) until the end of the study. Neither bandages nor medications were administered to avoid any interference with the inflammatory response. Horses' heart rate, respiratory rate, body temperature, and appetite were monitored twice daily to check for signs of discomfort.

Each fetlock was clinically evaluated on day 0 (before injection) then on days 1, 3, 7, 14, and 28. Clinical evaluation was performed blindly by the same operator (SJ). Firstly, the fetlock joint circumference was measured at the middle section of the proximal sesamoid bones and midmetacarpal/metatarsal area using a shaved skin landmark. Both measurements were taken in triplicate and averaged. Secondly, sensitivity to digital flexion tests and joint effusion were evaluated using a five-point scale from normal to severe (0: normal, 1: mild, 2: moderate, 3: substantial, and 4: severe). Presence of subcutaneous oedema was also evaluated and scored using the same five-point scale (see Table 1 and Supplementary 7). Each fetlock and metacarpal/tarsal area was photographed from the front and the side. The degree of lameness was graded on a scale of 0 to 5 in accordance with the lameness scale of the American Association of Equine Practitioners [31].

Ultrasonographic examination was performed on day 0 (before injection) then days 1, 3, 7, 14, and 28. The dorsal and collateral aspects of each fetlock were examined in transverse and longitudinal scans, using a 7.5 MHz linear transducer (Hitachi Medical Systems, Saint-Priest, France) (see Supplementary 5). Synovial fluid effusion was evaluated blindly by the same investigator (SJ) and graded with the same 5-point scale as for clinical evaluation (Table 2). In the case of a major inflammatory reaction leading to a marked discomfort of the horse (i.e., clinical or ultrasonographic grades ≥ 3/4 or ≥3/5 for lameness), investigators were allowed to administer NSAIDs to relieve pain and excessive inflammation. This deviation from the protocol excluded the horse from data analysis from the time point of drug administration onwards.

### 2.6. Synovial Fluid Analysis

Samples of synovial fluid (1 mL) were taken from each joint on day 0 (before injection) then days 7, 14, and 28 using the technique previously described for MSC injections (Supplementary 4). Sampling was not performed during the first week after MSC injection to avoid removing MSCs; MSCs have been tracked within synovial fluid for 12 weeks [32] and a pilot study that we performed confirmed a high number of MSCs in the sampling on days 1 and 3 but not from day 7 onwards. Approximately 0.3 mL of fluid was placed in an EDTA tube and total nucleated cell counts were directly measured using an automatic analysis system (Sysmex XN10, Sysmex Corporation, Japan) (cells/*μ*L). The remaining fluid was placed in a dry tube for centrifugation at 2500 × g for 10 minutes. After aqueous phase recovery, 20 *μ*L of the sample was placed on a refractometer (Zuzi series 300, Auxilab SL, Spain) to determine total protein concentration. The remainder was then stored at -80°C for ELISA analysis. Synovial fluid concentration of Prostaglandin E_2_ (PGE_2_) and C-terminal telopeptide of type II collagen (CTX-II) as markers of inflammation and cartilage degradation, respectively, was estimated by the use of commercially available high-sensitivity enzyme immunoassay kits (Prostaglandin E_2_ Parameter Assay Kit, R&D systems, USA; Serum Pre-Clinical CartiLaps® (CTX-II) EIA, Immunodiagnostic Systems Holdings PLC, UK) [33, 34].

### 2.7. Statistical Analysis

Statistical analysis was performed after the data has been visually assessed for normality. The mean and standard deviation of normally distributed outcomes and the median and quartiles of the others were calculated at each time point for each treatment and dosage groups (see Supplementary 3). To analyze the influence of the different dosages, the data from the placebo-treated joints were excluded and the data from all MSC types were pooled together. Similarly, to analyze the influence of the different treatments, the data from both dosages were pooled together. Two quantitative outcomes were normally distributed (total protein concentration and fetlock joint circumference) and two others were log-transformed to follow a normal distribution (PGE_2_ and CTX-II concentrations). Others outcomes (semiquantitative scores and total nucleated cell count) were not normally distributed and were turned into binary outcomes. A threshold of 200 cells/*μ*L consistent with the presence of OA was chosen for the total nucleated cell count, as described in published reports [34, 35]. For categorical outcomes, a binary classification was performed for the fetlocks according to the two following clinical categories:

- “Tolerated” when the assigned grades (sensitivity to digital flexion tests, joint effusion, degree of lameness, subcutaneous oedema, and synovial fluid effusion on ultrasound) were ≤1 out of 4 (or 5 for lameness). These signs were considered as acceptable signs of pain and inflammation
- “Not tolerated” when assigned grades were >1 out of 4 (or 5 for lameness). These were considered as excessive signs of pain and inflammation (see Supplementary 2)

When the outcome was binary, logistic regression models were performed using generalized estimating equations (GEE) to adjust for correlated repeated measurements within each horse [35]. When the outcome was quantitative and normally distributed, linear regression models were used also adjusting for correlated repeated measurements within each horse (by using GEE models with a normal link). In all models, horse was included as a random effect, and treatment (or dose) and time were included as fixed effects Reference values obtained on day 0 before intra-articular injections were excluded from analysis. Odds ratio (OR) with their 95% confidence interval (CI) was provided for binary outcomes, and differences in means (with their 95% CI) were provided for quantitative outcomes. SAS V9.4 (SAS Institute, Cary, NC) was used for all the GEE models (procedure GENMOD). Alpha type I error was set at 5%.

## 3. Results

### 3.1. MSC Isolation and Characterization

MSCs were successfully isolated and expanded in culture. Characterization by flow cytometry revealed the detection of CD29, CD44, and CD90 expression for all cell populations of each strain and the absence of CD45 and MHC class II expression. CD73 expression was donor-dependent, with expression by only part of the cell population. Similarly, CD105 was not detected for UCB-MSCs and was weakly expressed by BM-MSCs at P4 (data shown in other published reports [26, 29] and in Supplementary 6).

All the BM-MSC strains used in the present study had high proliferative capacity and possessed multipotency capacity to differentiate into osteoblasts, adipocytes, and chondrocytes, as previously described [28]. UCB-MSCs had the same capacities except for adipogenic differentiation. Indeed, no lipid droplets were detected in the cytoplasm of UCB-MSC by Oil Red O staining. Thus, UCB-MSCs had only partial mesenchymal lineage differentiation ability (data shown in other published reports [26, 29] and in Supplementary 6).

### 3.2. Intra-Articular Injections

**Figure 1:**
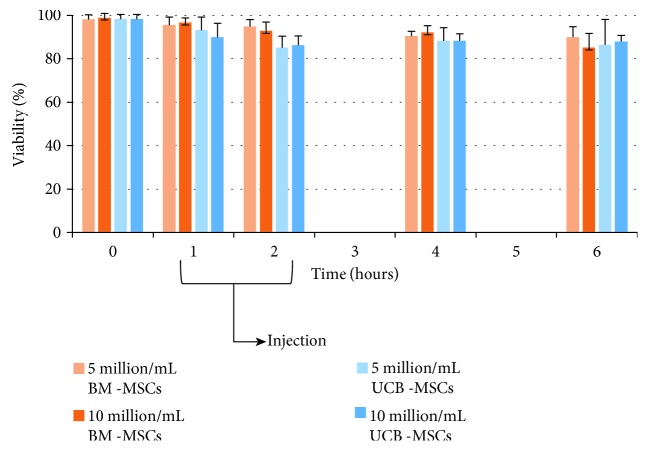
Mean viability of MSCs after culture and preparation for injection at 25°C.

Intra-articular injections were successfully performed in all fetlock joints. The cell lines from which each horse received allogeneic cells are available in Supplementary 2. Injections were made within 1 to 2 hours of MSC preparation. At the time of injection, mean viability of MSCs was between 96.6% (±2.2) and 92.7% (±4.2) for BM-MSCs and between 93.09% (±6.12) and 85.11% (±5.27) for UCB-MSCs (see Figure 1 and Supplementary 1).

### 3.3. Clinical Signs

**Figure 2:**
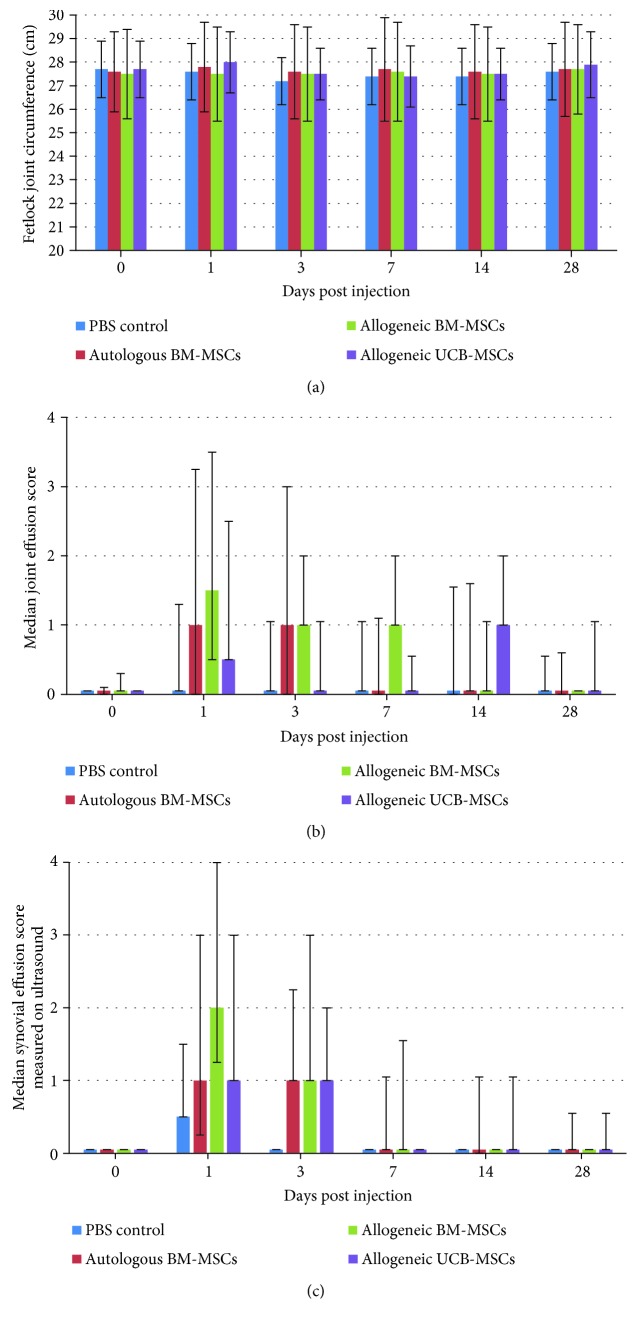
Results of clinical and ultrasound parameters of fetlock joint inflammation after intra-articular injection of MSCs or control PBS. (a) Fetlock joint circumference (in cm) expressed as the mean ± standard deviation. (b) Joint effusion score expressed as the median ± first and third quartiles. (c) Synovial effusion score measured by ultrasound expressed as the median ± first and third quartiles.

**Figure 3:**
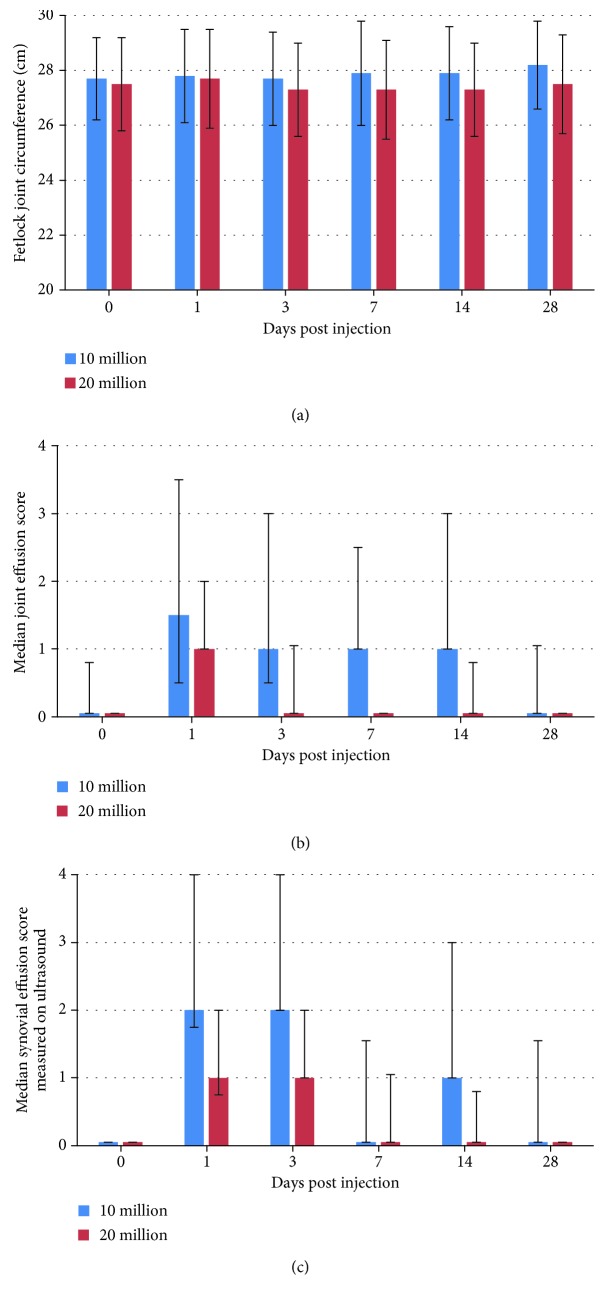
Results of clinical and ultrasound parameters of fetlock joint inflammation after intra-articular injection of 2 different dosages of BM-MSCs or UCB-MSCs. (a) Fetlock joint circumference (in cm) expressed as the mean ± standard deviation. (b) Joint effusion score expressed as the median ± first and third quartiles. (c) Synovial effusion score measured by ultrasound expressed as the median ± first and third quartiles.

**Figure 4:**
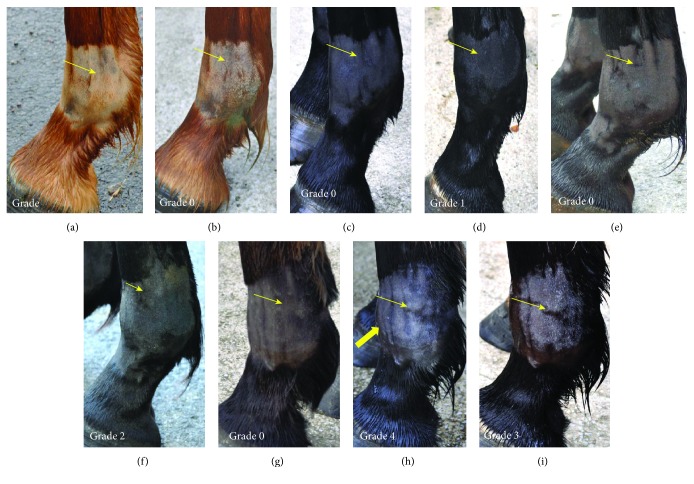
Photographs of the lateral aspect of the fetlock joint of 4 horses taken on day 0 (D0) before MSC injections (a, c, e, g), on day 1 (D1) after injection (b, d, f, h), and on day 7 (D7) after injection (i) showing the different grades of joint effusion observed. See the proximopalmar recess of the metacarpophalangeal joint (light arrow) and the distention of the dorsal recess of the joint (thick arrow). (a, b) Left front fetlock of horse 1 injected with allogeneic BM-MSCs showing grade 0 fetlock joint effusion on D0 and D1. (c, d) Left front fetlock of horse 3 injected with autologous BM-MSCs showing grade 0 fetlock joint effusion on D0 (c) and grade 1 on D1 (d). (e, f) Right front fetlock of horse 6 injected with allogeneic UCB-MSCs showing grade 0 fetlock joint effusion on D0 (e) and grade 2 on D1 (f). (g, h, i) Left front fetlock of horse 7 injected with allogeneic BM-MSCs showing grade 0 fetlock joint effusion on D0 (g), grade 4 on D1 (h), and grade 3 on D7.

**Figure 5:**
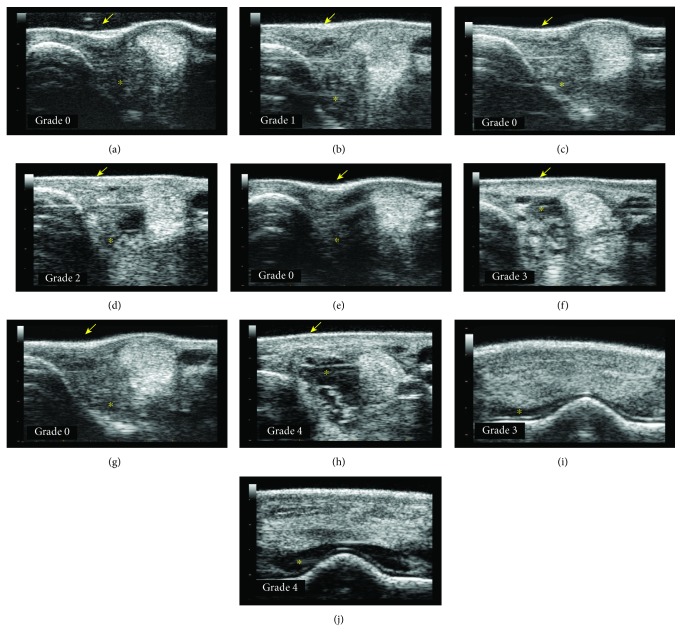
Transverse ultrasound images of the lateral aspect (a-h) and dorsal aspect (i, j) of the fetlock joint of 4 horses taken on day 0 (D0) before MSC injections (a, c, e, g), on day 1 (D1) after injection (b, d, h, j), and on day 3 (D3) after injection (f, i) showing the different grades of synovial effusion measured on ultrasound. Note the aspect of the skin (arrow) from concave to convex and the amount of fluid (^∗^) from mild to marked. (a, b) Right front fetlock of horse 1 injected with autologous BM-MSCs showing grade 0 synovial effusion on D0 (a) and grade 1 on D1 (b). (c, d) Left front fetlock of horse 10 injected with allogeneic UCB-MSCs showing grade 0 synovial effusion on D0 (c) and grade 2 on D1 (d). (e, f, i) Right front fetlock of horse 9 injected with allogeneic BM-MSCs showing grade 0 synovial effusion on D0 (e) and grade 3 on D3 (f, i). (g, h, j) Right front fetlock of horse 7 injected with autologous BM-MSCs showing grade 0 synovial effusion on D0 (g) and grade 4 on D1 (h, j).

Body temperature, heart rate, respiratory rate, and appetite remained normal for all the horses throughout the study. None of the 12 horses showed sensitivity to digital flexion tests throughout the study. Measurements of fetlock circumferences did not reveal any significant changes except for UCB-MSC-treated fetlocks that showed a significantly higher mean circumference than the control fetlocks (Table 3, Figures 2(a) and 3(a)). Four of the 12 horses showed mild to moderate unilateral lameness (grade 1-2/5) between day 1 and day 7 which was resolved by day 14. However, one of these horses (horse 7) showed concomitant grade 3 to 4/5 lameness on one limb on day 1 (the right hind limb injected with UCB-MSCs) with clear signs of intolerance over all 3 of its fetlocks injected with MSCs (clinical and ultrasonographic grades of synovitis ≥ 3) (Figures 4(g)–4(i), 5(g), 5(h), and 5(j); Supplementary 2). As bilateral lameness was suspected for the front limbs of this horse (limbs injected with allogeneic and autologous BM-MSCs) but could not be observed and graded, lameness data was not considered relevant in this study design and was excluded from statistical analysis. In addition, horse 7 received NSAIDs with 1.1 mg/kg intravenous flunixin meglumine (Finadyne, Intervet, Angers, France) every 12 h from day 1 (after clinical grading) until the resolution of clinical signs of discomfort on day 3. All data from this horse was thus also excluded from statistical analysis after day 1. The lameness grade went down to a 1/5 grade on day 3 and the horse was no longer lame on day 7.

Seventeen fetlocks from 8 horses injected with MSCs showed a >1/4 joint effusion grade on clinical examination (Supplementary 2). These fetlocks, classified in the “not tolerated” category, were significantly more frequent in allogeneic BM-MSC-treated fetlocks than in UCB-MSC-treated fetlocks but there were no significant differences between autologous and allogeneic groups. The statistical model used did not allow comparing each MSC-treated fetlock group to the control fetlocks because no control fetlock was classified in the “not tolerated” category during the study period, while each MSC-treated fetlock group showed at least 5 fetlocks classified in this category (Table 3, Figure 2(b)). Out of the 17 fetlocks classified in the “not tolerated” category, only 3, all of which were from horse 7 and had been injected with each MSC type, respectively, had substantial to severe joint effusion (grade ≥ 3/4). Finally, when considering joint effusion, the “not tolerated” fetlocks were significantly more frequent for the fetlocks receiving 10 million MSCs than for the fetlocks receiving 20 million MSCs (Table 3, Figure 3(b)).

### 3.4. Ultrasound Monitoring

According to the clinical follow-up of joint effusion, synovial fluid effusion measured by ultrasound revealed that 18 fetlocks injected with MSCs from 8 horses showed >1/4 effusion and were classified in the “not tolerated” category. These “not tolerated” fetlocks were also significantly more frequent in allogeneic BM-MSC-treated fetlocks compared to UCB-MSC-treated fetlocks, but they were also more frequent in allogeneic BM-MSCs compared to autologous MSCs (Table 3, Figure 2(c)). Again, no statistical comparison could be made between each MSC-treated fetlock group and the control fetlocks because no control fetlock was classified in the “not tolerated” category. Of the 18 “not tolerated” fetlocks, 6 from 4 horses (including 3 fetlocks from horse 7) showed substantial to severe ultrasound signs with ≥3/4 synovial fluid effusion. One of these fetlocks was injected with autologous BM-MSC, 3 fetlocks were injected with allogeneic BM-MSCs, and 2 fetlocks were injected with UCB-MSCs. Five fetlocks were injected with 10 million MSCs and one fetlock with 20 million. As for the corresponding clinical parameter, when considering synovial effusion on ultrasound, the “not tolerated” fetlocks were significantly more frequent for the fetlocks receiving 10 million MSCs than for the fetlocks receiving 20 million MSCs (Table 3, Figure 3(c)).

### 3.5. Synovial Fluid Analysis

**Figure 6:**
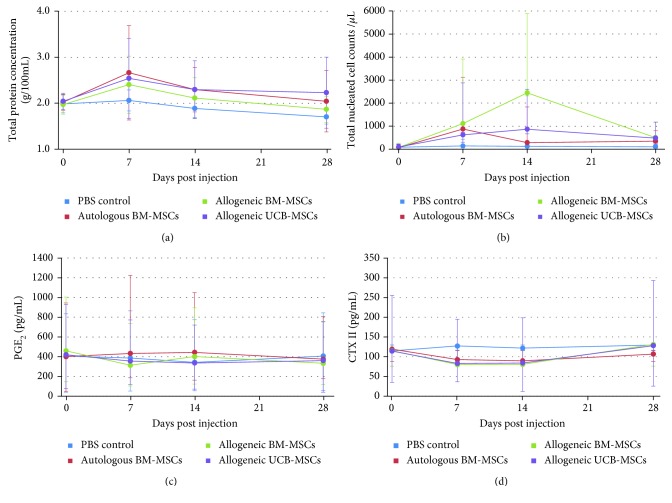
Results of synovial fluid analysis from fetlock joints after intra-articular injection of MSCs or PBS control. (a) Total protein concentration (g/100 mL) expressed as the mean ± standard deviation. (b) Total nucleated cell counts (per *μ*L) expressed as the median ± first and third quartiles. (c) PGE_2_ concentration (pg/mL) expressed as the median ± first and third quartiles. (d) CTX-II concentration (pg/mL) expressed as the median ± first and third quartiles.

**Figure 7:**
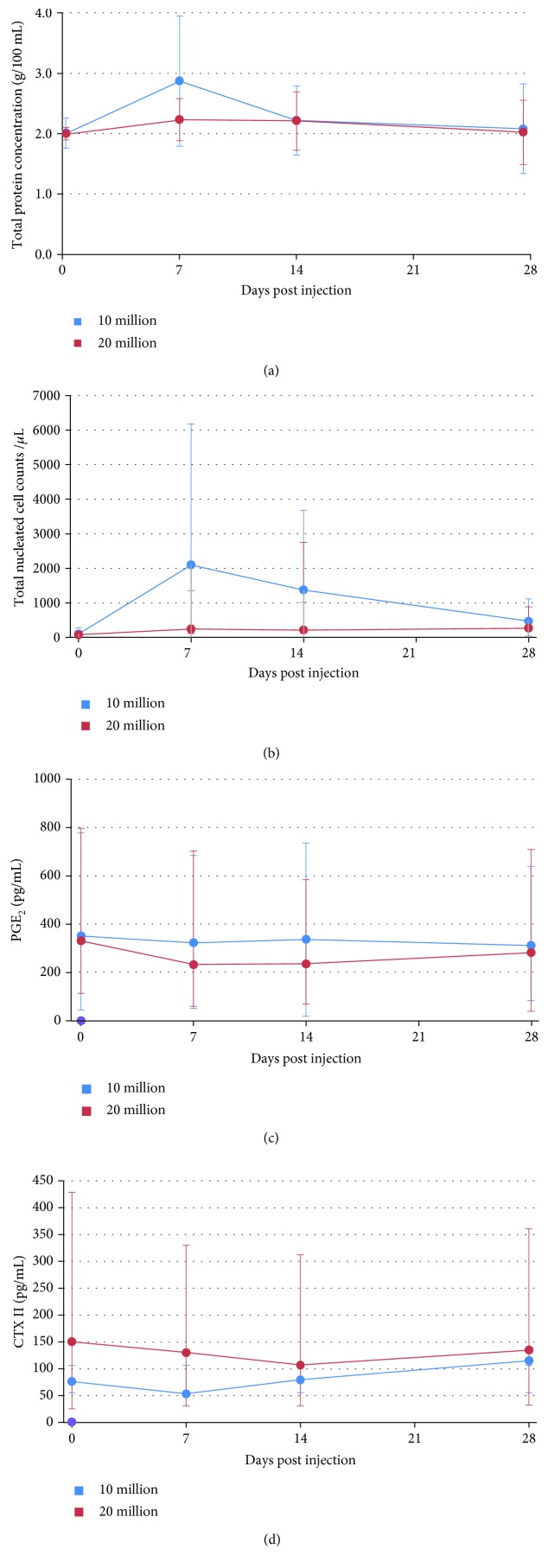
Results of synovial fluid analysis from fetlock joints after intra-articular injection of 2 different doses of BM-MSCs or UCB-MSCs. (a) Total protein concentration (g/100 mL) expressed as the mean ± standard deviation. (b) Total nucleated cell counts (per *μ*L) expressed as the median ± first and third quartiles. (c) PGE_2_ concentration (pg/mL) expressed as the median ± first and third quartiles. (d) CTX-II concentration (pg/mL) expressed as the median ± first and third quartiles.

Synovial fluid was successfully obtained at each time point for 32/48 fetlocks of the study and for at least 3 out of 4 time points for the others. When considering the frequency of fetlocks above the threshold of 200 nucleated cells and the mean total protein concentration, all MSC-injected fetlocks showed significantly higher levels than the PBS control-injected fetlocks (Table 3, Figures 6(a) and 6(b)), but there was no significant difference between MSC types. No significant differences were noted after injection of 10 million cells compared to 20 million cells regarding total protein levels but the frequency of fetlocks above the threshold of 200 nucleated cells was significantly higher for 10 million than for 20 million (Table 3, Figures 7(a) and 7(b)). Mean PGE_2_ concentrations were not significantly modified after joint injections throughout the study (Figures 6(c) and 7(c)). Conversely, mean CTX-II concentrations appeared to decrease after MSC injection compared to PBS control and baseline with significant differences between autologous BM-MSCs and PBS control and between UCB-MSCs and PBS control (Figure 6(d)). In addition, CTX-II concentrations appeared significantly lower in the 10 million group compared to the 20 million group (Table 3, Figure 7(d)).

## 4. Discussion

This study has two main findings. The first is the presence of significant differences between the inflammatory responses induced by allogeneic UCB-MSCs and allogeneic BM-MSCs when injected into the healthy joints of the same individuals, but only when considering synovial effusion (measured both clinically and by ultrasound), not regarding other local clinical and ultrasound signs as well as synovial fluid parameters. Allogeneic sources were also compared to autologous BM-MSCs and significant differences were noted but only between BM sources for synovial effusion measured by ultrasound and total protein concentration with higher levels after allogeneic BM-MSC injections. This confirms one previously reported finding [17] but contradicts the results of other safety studies where no differences were seen after a single injection [15, 20]. The second finding is that injection of 10 million MSCs per joint induces significantly more synovial effusion and increase in total nucleated cell counts than the injection of 20 million MSCs. These results supporting the use of higher MSC doses are not in accordance with recent human clinical preliminary trials in the treatment of knee OA, where significant clinical improvement was obtained after intra-articular administration of the lowest dose (2 million) of autologous adipose-derived MSCs [36]. The doses of MSCs evaluated in this study were within the ranges of the doses recommended and routinely used in clinical equine practice [32] or for experimental and clinical trials on horses [9, 15–17, 20], even if there is still no consensus on the appropriate dosage [23]. The dosage results of this study should however to be tempered in view of the individual variability of the inflammatory responses observed. Indeed, it would have been more reliable to evaluate the different dosages on the same horses.

PBS injections were used to monitor the response of the joint to arthrocentesis and to the transport medium. As previously reported [15–17], results from this study tend to confirm that the intra-articular injection of native MSCs in healthy joints, whether autologous or allogeneic, seems to induce a transient synovial fluid effusion observed on Figures 2(b) and 2(c) that is not observed after an identical injection of saline control. Unfortunately, no statistical comparison could be made on these 2 parameters. Nevertheless, a significant increase in total nucleated cell counts and total protein concentration was noted in all MSC-treated fetlock groups compared to the control fetlock group with similar patterns to those previously reported [17] from day 7 to day 28, with values returning to baseline by day 28. No significant differences in PGE_2_ concentrations were observed between MSC types and the placebo throughout the study, suggesting that no major deleterious inflammatory reaction occurs after MSC injections. Interestingly, UCB-MSCs induced a significant decrease in CTX-II concentration compared to PBS control. A similar decrease was also observed in CTX-II concentration, a biomarker of cartilage degradation [34, 37], after autologous BM-MSC injections, but to a lesser extent. These data could reveal a potential chondroprotective effect of MSCs that is more marked with UCB-MSCs. This effect seems to be limited in time by a return to baseline values of CTX-II concentration by day 28 for all MSC types, highlighting the potential interest of performing repeated injections of MSCs for a therapeutic use as previously suggested [15, 20]. Some missing values in synovial fluid parameters might however have influenced the results. In addition, the synovial fluid results of the first week are certainly underestimated because of the absence of sampling on days 1 and 3. Indeed, the peaks in synovial fluid parameters generally occurred on day 1 and were predominantly resolved between days 3 and 10 in previous studies [15–17]. The same studies also reported that synovial fluid sampling combined with saline injection caused a nonsignificant transient increase in synovial fluid cell counts and total protein concentration the day after sampling that was unrelated to the administration of MSCs. We were unable to verify this in our study due to the absence of sampling the day after MSC injection.

As regards the clinical parameters, the most reactive parameter after injections was synovial effusion. Of the 36 fetlocks treated with MSCs, 17 (47%) had been assigned a grade > 1 and were classified in the “*not tolerated*” category, which might be a concern for owners and thus constitute a limiting factor for the use of this therapy on privately owned animals. Nevertheless, significant differences with the placebo group could not have been assessed and synovial effusion was moderate for most of the fetlocks (12/17) and decreased by the end of the study, when it was classified in the “*tolerated*” category except in 3/17 fetlocks, for which it remained stable. Concerning the lameness grades, the study design with concurrent injections on the same horses unfortunately made it impossible to draw conclusions about this parameter which may have been underestimated, as bilateral lameness could not be evaluated correctly. This limitation of the study led us to exclude lameness data from the analysis. To improve pain evaluation and assess bilateral lameness, it would be interesting to measure stride length in future studies.

The results of the present study suggest that intra-articular injection of a same line of MSCs on healthy joints induces a variable degree of local inflammatory response according to the individual. Even if only one of the 12 horses (8.3%) in this study (horse 7) exhibited severe signs of pain and inflammation, this kind of reaction could be observed on any client-owned animal participating in a clinical trial. Growing evidence indicates that future clinical applications of stem cell therapies will not aim at injecting MSCs alone or simply diluted in PBS but rather suggests that MSCs would gain therapeutic interest if combined with extracellular matrix substitutes like hydrogels in order to enhance their viability and metabolic activity [23, 38]. Recent studies even indicate that the therapeutic activities of MSCs could be achieved by injecting solely the bioactive secretory factors of MSCs, called secretome, suggesting a future cell-free/cell-based strategy [23, 39, 40]. Although these new strategies might improve the safety of MSCs, it is still crucial to understand the behavior of cells injected alone to optimize recipient responses.

The cause of the inflammatory responses observed is still not perfectly understood. Sepsis was excluded based on synovial fluid analysis and clinical evolution (see Supplementary 2). It is important to note that total protein concentration and total nucleated cell counts remained under the admitted thresholds for septic arthritis [41] except for one fetlock of horse 9 which approached these values. Indeed, a 5 g/L total protein concentration and 23945 cells/*μ*L were measured in the synovial fluid of its fetlock seven days after injection with autologous BM-MSCs. No associated clinical signs were observed, and values progressively returned to baseline values by the end of the study without any treatment. The clinical evolution in horse 7, which showed severe signs of synovial effusion, was spontaneously favorable without any other treatment than 3 days of anti-inflammatory drugs. In addition, other horses from the study received the same pools of cells as horse 7 and did not show any local inflammatory response. The fact that three joints from horse 7 treated with different types of MSCs reacted in the same way points to the likely individual predisposition of this horse.

Even if no dramatic increase in total nucleated cell counts was measured and every line of injected MSCs was tested for the absence of MHC class II, the signs observed could be explained by an immune reaction. Indeed, it has been demonstrated that equine allogeneic MSCs are capable of eliciting antibody responses *in vivo* with individual variations in strength and timing and independently of MSC MHC class II expression [19]. The authors of that study suggest that both donor and recipient horses should be screened for MHC compatibility before MSC administration. The access to microsatellite typing is however limited and was unfortunately not available in our laboratory. Nevertheless, the occurrence of such MSC immunogenicity is not supported by the finding that 10 million cells result in greater inflammation than 20 million, as fewer reactions with an increased cell number would rather suggest an increase in immunomodulation. Unfortunately, the immunomodulation potential of the MSCs *in vitro* was not measured in this study.

Fetal bovine serum should also be considered as a potential cause of the different reactions observed in the horses, despite the effort that had been made to remove all the culture media. FBS was used in our culture and a residual contamination with xenoproteins could have occurred, as highlighted by a recent study [20] in which FBS intracellular xenogen-contaminated autologous MSCs induced a significant immune reaction compared to FBS-depleted MSCs. However, this significant reaction was not noted after a first injection but only after a primed injection, in contrast with the results of the present study. Two other characteristics of the reactions observed in our study do not support the hypothesis of humoral antibody responses. First, the reactions were not only observed after allogeneic injections but also after autologous MSC injections. Similar inflammatory responses have already been reported after intra-articular injections of autologous BM-MSCs [9, 15]. MHC expression is known to be dynamic and dependent on environmental factors like the presence of interferon gamma for MHC class II [18, 42]. It is possible that class I or class II MSC MHC can change their expression profile after injection into the fetlock joints if proinflammatory cytokines are already present in the synovial fluid. Another possibility could be the cross-reactive antibody responses directed against epitopes of the MHC class I antigens, as described for high-titered anti-MHC antiserum [19]. The second characteristic that does not support the theory of humoral antibody responses is that reactions started occurring from 24 hours after injection, while humoral antibody responses tend to occur 7 to 14 days after injections [19]. This time sequence rather points to the occurrence hypersensitivity reactions or reactive arthritis. Reactive arthritis has already been reported after the intra-articular injection of chemical or biologically active substances like polysulfated glycosaminoglycans [43] or hyaluronic acid. Sensitivity to paracrine molecules secreted by the stem cells should be considered, as it is now commonly accepted that stem cells can have variable behaviors depending on the environment in which they evolve [6, 44]. Osmotic shock or massive MSC death could also be responsible for such reactive arthritis, although results of a recent *in vitro* study report an increased viability of BM-MSC when cultured in 100% synovial fluid up to 72 h, compared to control media culture [45]. The hypothesis of cell death is still supported by the differences observed in inflammatory responses between the two dosages evaluated in our study, with fewer reactions when a higher cell density is used. Indeed, it has been demonstrated that chondrocytes in dense suspension culture survive in serum-free culture medium because they secrete low molecular mass compounds that support their own viability [9].

This study has two main limitations. First, the design implied a small number of horses in which several MSC sources were injected within the same horse. Therefore, the results of the statistical analysis should be taken with caution given that and because no correction for multiple testing has been performed. Another consequence of the study design is that an objective evaluation of the degree of lameness of each limb was not possible, meaning that this parameter could not be considered in the data analysis. Finally, this study design could potentially induce a systemic effect and affect the responses seen within the individual. The immunomodulatory capabilities of the MSCs injected were not measured in this study. Although the immunomodulatory properties and immunogenicity of MSCs have been widely reported and are already used in clinical settings [7, 46–48], the mechanisms underlying these effects have not been clearly defined [46, 48], and to our knowledge, the studies performed to date focused on general infusion of MSCs [49], but no study to date has focused on the systemic effect of MSCs after a local intra-articular administration. A reduction in trafficking of MSCs has been reported due to their size, which promotes passive cell entrapment [49]. This might be the case for the joint, where the synovial membrane constitutes a blood-joint barrier and this limits exchanges of the synovial fluid and its content between the joint cavity and the bloodstream [50]. A systemic effect could have also occurred because of MSC-secreting factors or other soluble factors secreted in the presence of MSCs [46]. However, this also seems unlikely considering the results for horse 7, in which all joints reacted at the same time.

The second limitation of this study is the absence of MHC haplotype analysis and immunophenotyping of donor and recipient horses to know if pairs were MHC matches or mismatches. If available, such analysis would be beneficial to improve the understanding of the mechanisms leading to the occurrence of horse-dependent adverse reactions. To confirm or disprove the occurrence of an immune reaction, it would also have been interesting to perform repeated injections.

## 5. Conclusions

This study has shown that injections into the healthy joints of the same individuals of allogeneic BM-MSCs induce significantly more synovial effusion of the joint (measured both clinically and by ultrasound) compared to allogeneic UCB-MSCs without significant differences within the synovial fluid parameters. A significant transient increase in total protein concentration and nucleated cell counts with individual variations in strength was observed following MSC injections whatever the tissue source. Despite this elevation of synovial fluid parameters, CTX-II and PGE_2_ concentrations show that MSCs seemed to have a protective effect on cartilage degradation rather than being deleterious. In the present study, this effect was more pronounced after injection of UCB-MSCs. This study also revealed that injection of 10 million MSCs per joint induces significantly more clinical and ultrasound signs of synovial effusion and increase in total nucleated cell count than the injection of 20 million MSCs. However, the results of this study should be taken with caution given the small number of horses, the concurrent administration of multiple MSC sources on the same individuals, and the lack of information regarding donor and recipient matching, lameness grades, and synovial fluid analysis during the first week of study. As a step toward a safer use of stem cell therapies in the future, either combined to biomaterials as vehicles or used for their secretome, this study nevertheless highlights the need for further investigation to better understand the causes of reactive arthritis induced by intra-articular injection of MSCs on the horse in order to find new preventive methods. The results of this study will also be useful in human medicine, as the horse is recognized as a reliable model for human osteoarthritis.

## Figures and Tables

**Table 1:** Fetlock joint effusion grading system.

Score		Physical criteria
0	Normal	Concave aspect of the proximopalmar recess of the metacarpo(tarso)phalangeal joint. No lateral swelling when a medial pressure is applied on the recess.
1	Mild	Flat aspect of the proximopalmar recess of the metacarpo(tarso)phalangeal joint. Mild lateral swelling when a medial pressure is applied on the recess.
2	Moderate	Convex aspect of the proximopalmar recess of the metacarpo(tarso)phalangeal joint. Lateral swelling easy to obtain when a medial pressure is applied on the recess.
3	Substantial	Convex aspect of the proximopalmar recess of the metacarpo(tarso)phalangeal joint exceeding the suspensory ligament branches (third interosseous muscle). Soft consistency of the recess on palpation.
4	Severe	Convex aspect of the proximopalmar recess of the metacarpo(tarso)phalangeal joint exceeding the suspensory ligament branches (third interosseous muscle) with a hard consistency of the recess on palpation indicating synovial pressure. Synovial distension of the dorsal recess of the joint.

**Table 2:** Synovial fluid effusion grading system measured by ultrasound.

Score		Ultrasound criteria
0	Normal	Mild amount of fluid in the proximopalmar recess of the metacarpo(tarso)phalangeal joint. Concave aspect of the skin. No motion of the fluid when pressing on the recess.
1	Mild	Mild amount of fluid in the proximopalmar recess of the metacarpo(tarso)phalangeal joint. Concave to flat aspect of the skin. Motion of the fluid when pressing on the recess.
2	Moderate	Moderate amount of fluid in the proximopalmar recess of the metacarpo(tarso)phalangeal joint. Convex aspect of the skin. Motion of the fluid when pressing on the recess.
3	Substantial	Substantial amount of fluid in the proximopalmar recess of the metacarpo(tarso)phalangeal joint and mild amount of fluid on the dorsal recess of the joint. Convex aspect of the skin.
4	Severe	Marked amount of fluid in the proximopalmar recess of the metacarpo(tarso)phalangeal joint and substantial amount of fluid on the dorsal recess of the joint with synovial pressure. Convex aspect of the skin.

**Table 3:** Differences in means (95% CI; *p*) or odds ratio (95% CI; *p*) of the outcomes used to compare treatment and dosage groups.

	Quantitative outcomes: difference in means (95% CI); *p*				Binary outcomes: OR (95% CI); *p*		
	Fetlock circumference	Total protein	Log10 (PGE_2_)	Log10 (CTX-II)	Total nucleated cell counts	Joint effusion	Synovial fluid effusion
Autologous BM-MSCs vs. placebo	0.2 (-0.7 to 1.1); 0.66	**0.5 (0.1** to **0.8)**; **0.006** ^∗^	-0.07 (-0.3 to 0.1); 0.50	**-0.1 (-0.2** to **0.03)**; **0.008** ^∗^	**6.7 (1.5** to **29.8)**; **0.01** ^∗^	NC	NC
Allogenic BM-MSCs vs. placebo	0.1 (-0.8 to 1); 0.77	**0.2 (0.05** to **0.4)**; **0.01** ^∗^	-0.15 (-0.4 to 0.08); 0.20	-0.1 (-0.2 to 0.01); 0.06	**8.0 (1.9** to **33.7)**; **0.005** ^∗^	NC	NC
Allogenic UCB-MSCs vs. placebo	**0.2 (0.1** to **0.3)**; **0.006** ^∗^	**0.5 (0.1** to **0.8)**; **0.005** ^∗^	-0.08 (-0.2 to 0.04); 0.20	**-0.2 (-0.2** to **-0.1)**; **<0.0001** ^∗^	**12 (1.7** to **83.5)**; **0.01** ^∗^	NC	NC
Autologous vs. allogenic BM-MSCs	0.07 (-0.1 to 0.2); 0.38	0.2 (-0.002 to 0.4); 0.052	0.08 (-0.05 to 0.2); 0.24	-0.03 (-0.1 to 0.1); 0.67	0.8 (0.3 to 2.3); 0.74	0.6 (0.3 to 1.1); 0.12	**0.5 (0.3** to **0.8)**; **0.004** ^∗^
Autologous BM vs. allogenic UCB-MSCs	0.001 (-1 to 1); 0.10	-0.01 (-0.3 to 0.3); 0.94	0.001 (-0.2 to 0.2); 0.99	0.02 (-0.1 to 0.1); 0.65	0.6 (0.1 to 2.5); 0.44	0.9 (0.3 to 1.9); 0.75	1.1 (0.6 to 2); 0.68
Allogenic BM vs. allogenic UCB-MSCs	-0.07 (-1 to 0.9); 0.88	-0.2 (-0.5 to 0.03); 0.08	-0.07 (-0.3 to 0.1); 0.50	0.04 (-0.04 to 0.1); 0.30	0.7 (0.2 to 2.6); 0.55	**1.5 (1** to **2.4)**; **0.049** ^∗^	**2.2 (1.3** to **3.9)**; **0.004** ^∗^
10 vs. 20 million cells	0.5 (-0.9 to 2); 0.48	0.2 (-0.1 to 0.5); 0.26	0.1 (-0.1 to 0.3); 0.2	**-0.27 (-0.4** to **0.1)**; **0.003** ^∗^	**4.2 (1.5** to **11.5)**; **0.005** ^∗^	**7.0 (1.7** to **28.3)**; **0.006** ^∗^	**9.4 (1.9** to **46)**; **0.006** ^∗^

OR: odds ratio; CI: confidence interval; NC: not comparable with the model used; ^∗^ *p* < 0.05.

## Data Availability

The database used to support the findings of this study is included within the supplementary information materials.

## Supplementary Materials

Supplementary 1 Dataset S1: viability tests of MSC results.

Supplementary 2 Dataset S2: scores and values of clinical, ultrasound, and synovial fluid parameters.

Supplementary 3 Dataset S3: mean (standard deviation) or median (first quartile, third quartile) scores and values of clinical, ultrasound, and synovial fluid parameters within the 48 fetlocks from day 0 to day 28.

Supplementary 4 Figure S1: synovial fluid sampling followed by intra-articular injection of MSCs or placebo in a metatarsophalangeal joint of one horse using a lateral approach on the flexed limb.

Supplementary 5 Figure S2: ultrasound technique and reference images.

Supplementary 6 Figure S3: characterization of equine bone marrow MSCs and umbilical cord blood MSCs.

Supplementary 7 Table S1: distribution of MSC treatments. Table S2: quality control of MSCs. Table S3: sensitivity to digital flexion tests grading system. Table S4: subcutaneous oedema grading system.
