# Supplementary material for: Intra-Articular Injection of 2 Different Dosages of Autologous and Allogeneic Bone Marrow- and Umbilical Cord-Derived Mesenchymal Stem Cells Triggers a Variable Inflammatory Response of the Fetlock Joint on 12 Sound Experimental Horses
Source: Stem Cells Int. 2019 May 2;2019:9431894. doi: 10.1155/2019/9431894 (PMC6525957; doi:10.1155/2019/9431894)

Concentration = 5 million cells per mL of PBS; T°C = 25

	UCB-01				BM-07			
	Total number of cells	Total number of dead cells	% mortality	% viability	Total number of cells	Total number of dead cells	% mortality	% viability
T= 0h	91	0	0,00	100,00	100	1	1,00	99,00
T= 1h	149	12	8,05	91,95	100	0	0,00	100,00
T= 2h	82	9	10,98	89,02	100	2	2,00	98,00
T= 4h	99	8	8,08	91,92	100	12	12,00	88,00
T= 6h	80	12	15,00	85,00	110	15	13,64	86,36

	UCB-02				BM-08			
	Total number of cells	Total number of dead cells	% mortality	% viability	Total number of cells	Total number of dead cells	% mortality	% viability
T= 0h	127	0	0,00	100,00	103	6	5,83	94,17
T= 1h	77	4	5,19	94,81	101	5	4,95	95,05
T= 2h	82	8	9,76	90,24	100	4	4,00	96,00
T= 4h	127	10	7,87	92,13	100	10	10,00	90,00
T= 6h	89	8	8,99	91,01	97	4	4,12	95,88

	UCB-03				BM-09			
	Total number of cells	Total number of dead cells	% mortality	% viability	Total number of cells	Total number of dead cells	% mortality	% viability
T= 0h	80	0	0,00	100,00	100	0	0,00	100,00
T= 1h	89	2	2,25	97,75	101	5	4,95	95,05
T= 2h	92	15	16,30	83,70	100	3	3,00	97,00
T= 4h	68	3	4,41	95,59	100	9	9,00	91,00
T= 6h	91	7	7,69	92,31	79	12	15,19	84,81

	UCB-04				BM-10			
	Total number of cells	Total number of dead cells	% mortality	% viability	Total number of cells	Total number of dead cells	% mortality	% viability
T= 0h	92	2	2,17	97,83	113	0	0,00	100,00
T= 1h	16	0	0,00	100,00	117	7	5,98	94,02
T= 2h	84	12	14,29	85,71	110	4	3,64	96,36
T= 4h	78	13	16,67	83,33	104	6	5,77	94,23
T= 6h	73	6	8,22	91,78	114	5	4,39	95,61

	UCB-05				BM-11			
	Total number of cells	Total number of dead cells	% mortality	% viability	Total number of cells	Total number of dead cells	% mortality	% viability
T= 0h	83	3	3,61	96,39	100	2	2,00	98,00
T= 1h	68	10	14,71	85,29	100	1	1,00	99,00
T= 2h	90	12	13,33	86,67	160	13	8,13	91,88
T= 4h	64	12	18,75	81,25	156	18	11,54	88,46
T= 6h	71	5	7,04	92,96	135	18	13,33	86,67

	UCB-06				BM-12			
	Total number of cells	Total number of dead cells	% mortality	% viability	Total number of cells	Total number of dead cells	% mortality	% viability
T= 0h	81	4	4,94	95,06	131	4	3,05	96,95
T= 1h	80	9	11,25	88,75	154	16	10,39	89,61
T= 2h	81	20	24,69	75,31	149	15	10,07	89,93
T= 4h	41	6	14,63	85,37	140	13	9,29	90,71
T= 6h	26	9	34,62	65,38	141	14	9,93	90,07

Concentration = 10 million cells per mL of PBS; T°C = 25

	UCB-01				BM-01			
	Total number of cells	Total number of dead cells	% mortality	% viability	Total number of cells	Total number of dead cells	% mortality	% viability
T= 0h	91	0	0,00	100,00	125	3	2,40	97,60
T= 1h	57	2	3,51	96,49	100	4	4,00	96,00
T= 2h	66	14	21,21	78,79	140	4	2,86	97,14
T= 4h	79	8	10,13	89,87	100	6	6,00	94,00
T= 6h	66	5	7,58	92,42	100	6	6,00	94,00

	UCB-02				BM-02			
	Total number of cells	Total number of dead cells	% mortality	% viability	Total number of cells	Total number of dead cells	% mortality	% viability
T= 0h	127	0	0,00	100,00	100	2	2,00	98,00
T= 1h	46	3	6,52	93,48	130	3	2,31	97,69
T= 2h	55	2	3,64	96,36	131	14	10,69	89,31
T= 4h	40	4	10,00	90,00	133	14	10,53	89,47
T= 6h	44	8	18,18	81,82	141	15	10,64	89,36

	UCB-06				BM-03			
	Total number of cells	Total number of dead cells	% mortality	% viability	Total number of cells	Total number of dead cells	% mortality	% viability
T= 0h	80	0	0,00	100,00	131	4	3,05	96,95
T= 1h	95	6	6,32	93,68	120	2	1,67	98,33
T= 2h	85	13	15,29	84,71	122	13	10,66	89,34
T= 4h	55	6	10,91	89,09	130	12	9,23	90,77
T= 6h	71	6	8,45	91,55	113	7	6,19	93,81

	UCB-14				BM-04			
	Total number of cells	Total number of dead cells	% mortality	% viability	Total number of cells	Total number of dead cells	% mortality	% viability
T= 0h	92	2	2,17	97,83	100	1	1,00	99,00
T= 1h	96	18	18,75	81,25	100	0	0,00	100,00
T= 2h	104	14	13,46	86,54	100	5	5,00	95,00
T= 4h	71	8	11,27	88,73	100	13	13,00	87,00
T= 6h	81	10	12,35	87,65	100	22	22,00	78,00

	UCB-15				BM-05			
	Total number of cells	Total number of dead cells	% mortality	% viability	Total number of cells	Total number of dead cells	% mortality	% viability
T= 0h	83	3	3,61	96,39	103	6	5,83	94,17
T= 1h	105	10	9,52	90,48	121	5	4,13	95,87
T= 2h	77	8	10,39	89,61	104	11	10,58	89,42
T= 4h	68	12	17,65	82,35	100	12	12,00	88,00
T= 6h	49	7	14,29	85,71	100	7	7,00	93,00

	UCB-17				BM-06			
	Total number of cells	Total number of dead cells	% mortality	% viability	Total number of cells	Total number of dead cells	% mortality	% viability
T= 0h	81	4	4,94	95,06	100	0	0,00	100,00
T= 1h	128	21	16,41	83,59	127	8	6,30	93,70
T= 2h	70	13	18,57	81,43	100	14	14,00	86,00
T= 4h	59	6	10,17	89,83	100	5	5,00	95,00
T= 6h	61	7	11,48	88,52	95	16	16,84	83,16

Mean viability (%) - 5.10^6 cells/mL

Time (hours)	5 million/mL BM - MSCs		5 million/mL UCB - MSCs	
	Mean	SD	Mean	SD
**0**	98,02	2,22	98,21	2,19
**1**	95,45	3,74	93,09	6,12
**2**	94,86	3,20	85,11	5,27
**4**	90,40	2,23	88,26	6,04
**6**	89,90	4,84	86,41	11,69

Mean viability (%) - 10.10^6 cells/mL

Time (hours)	10 million/mL BM - MSCs		10 million/mL UCB - MSCs	
	Mean	SD	Mean	SD
**0**	98,87	2,00	98,21	2,19
**1**	96,57	2,21	89,83	6,51
**2**	92,71	4,17	86,24	4,24
**4**	92,00	3,22	88,31	3,18
**6**	85,05	6,61	87,95	2,78

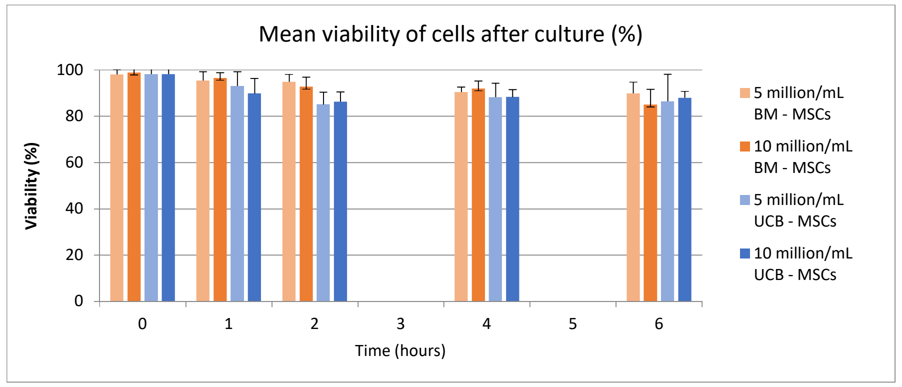

Supplement: Supplementary 1 — Dataset S1: viability tests of MSC results. [file 9431894.f1.pdf]
