# Supplementary material for: Intra-Articular Injection of 2 Different Dosages of Autologous and Allogeneic Bone Marrow- and Umbilical Cord-Derived Mesenchymal Stem Cells Triggers a Variable Inflammatory Response of the Fetlock Joint on 12 Sound Experimental Horses
Source: Stem Cells Int. 2019 May 2;2019:9431894. doi: 10.1155/2019/9431894 (PMC6525957; doi:10.1155/2019/9431894)
Supplement: Supplementary 3 — Dataset S3: mean (standard deviation) or median (first quartile, third quartile) scores and values of clinical, ultrasound, and synovial fluid parameters within the 48 fetlocks from day 0 to day 28. [file 9431894.f3.pdf]

Mean (standard deviation) or Median (first quartile, third quartile) scores and values of clinical and ultrasound parameters

Treatment	Day	Fetlock circumference	Lameness (grade/5)	Flexion sensitivity	Joint effusion	Subcutaneous oedema	Synovial fluid effusion
Placebo	0	27,7 (1.2)	0 (0-0)	0 (0-0)	0 (0-0)	0 (0-0)	0 (0-0)
	1	27,6 (1.2)	0 (0-0)	0 (0-0)	0 (0-0.25)	0 (0-0)	0.5 (0-1)
	3	27,2 (1)	0 (0-0)	0 (0-0)	0 (0-0)	0 (0-0)	0 (0-0)
	7	27,4 (1.2)	0 (0-0)	0 (0-0)	0 (0-0)	0 (0-0)	0 (0-0)
	14	27,4 (1.2)	0 (0-0)	0 (0-0)	0 (0-0)	0 (0-0)	0 (0-0)
	28	27,6 (1.2)	0 (0-0)	0 (0-0)	0 (0-0)	0 (0-0)	0 (0-0)
Autologous BM-MSCs	0	27,6 (1.7)	0 (0-0)	0 (0-0)	0 (0-0)	0 (0-0)	0 (0-0)
	1	27,8 (1.9)	0 (0-0)	0 (0-0)	1 (0-1.25)	0 (0-0)	1 (0.75-2)
	3	27,6 (2)	0 (0-0)	0 (0-0)	1 (0-1)	0 (0-0)	1 (0-1.25)
	7	27,7 (2.2)	0 (0-0)	0 (0-0)	0 (0-1)	0 (0-0)	0 (0-1)
	14	27,6 (2)	0 (0-0)	0 (0-0)	0 (0-1.5)	0 (0-0)	0 (0-1)
	28	27,7 (2)	0 (0-0)	0 (0-0)	0 (0-0.5)	0 (0-0)	0 (0-0.5)
Allogeneic BM-MSCs	0	27,5 (1.9)	0 (0-0)	0 (0-0)	0 (0-0.25)	0 (0-0)	0 (0-0)
	1	27,5 (2)	0 (0-0)	0 (0-0)	1.5 (1-2)	0 (0-0)	2 (0.75-2)
	3	27,5 (2)	0 (0-0)	0 (0-0)	1 (0-1)	0 (0-0)	1 (0-2)
	7	27,6 (2.1)	0 (0-0)	0 (0-0)	1 (0-1)	0 (0-0)	0 (0-1.5)
	14	27,5 (2)	0 (0-0)	0 (0-0)	0 (0-1)	0 (0-0)	0 (0-1)
	28	27,7 (1.9)	0 (0-0)	0 (0-0)	0 (0-0)	0 (0-0)	0 (0-0)
Allogeneic UCB-MSCs	0	27,7 (1.2)	0 (0-0)	0 (0-0)	0 (0-0)	0 (0-0)	0 (0-0)
	1	28 (1.3)	0 (0-0)	0 (0-0)	0.5 (0-2)	0 (0-0)	1 (0-2)
	3	27,5 (1.1)	0 (0-0)	0 (0-0)	0 (0-1)	0 (0-0)	1 (0-1)
	7	27,4 (1.3)	0 (0-0)	0 (0-0)	0 (0-0.5	0 (0-0)	0 (0-0)
	14	27,5 (1.1)	0 (0-0)	0 (0-0)	1 (0-1)	0 (0-0)	0 (0-1)
	28	27,9 (1.4)	0 (0-0)	0 (0-0)	0 (0-1)	0 (0-0)	0 (0-0.5)

Mean (standard deviation) or Median (first quartile, third quartile) scores and values of clinical and ultrasound parameters

Dosage	Day	Fetlock circumference (mean value)	Lameness (grade/5)	Flexion sensitivity	Joint effusion	Subcutaneous oedema	Synovial fluid effusion
10 million	0	27,7 (1,5)	0 (0-0)	0 (0-0)	0 (0-0.75)	0 (0-0)	0 (0-0)
	1	27,8 (1,7)	0 (0-0)	0 (0-0)	1.5 (1-2)	0 (0-0)	2 (0.25-2)
	3	27,7 (1,7)	0 (0-0)	0 (0-0)	1 (0.5-2)	0 (0-0)	2 (0-2)
	7	27,9 (1,9)	0 (0-0)	0 (0-0)	1 (0-1.5)	0 (0-0)	0 (0-1.5)
	14	27,9 (1,7)	0 (0-0)	0 (0-0)	1 (0-2)	0 (0-0)	1 (0-2)
	28	28,2 (1,6)	0 (0-0)	0 (0-0)	0 (0-1)	0 (0-0)	0 (0-1.5)
20 million	0	27,5 (1,7)	0 (0-0)	0 (0-0)	0 (0-0)	0 (0-0)	0 (0-0)
	1	27,7 (1,8)	0 (0-0)	0 (0-0)	1 (0-1)	0 (0-0)	1 (0.25-1)
	3	27,3 (1,7)	0 (0-0)	0 (0-0)	0 (0-1)	0 (0-0)	1 (0-1)
	7	27,3 (1,8)	0 (0-0)	0 (0-0)	0 (0-0)	0 (0-0)	0 (0-1)
	14	27,3 (1,7)	0 (0-0)	0 (0-0)	0 (0-0.75)	0 (0-0)	0 (0-0.75)
	28	27,5 (1,8)	0 (0-0)	0 (0-0)	0 (0-0)	0 (0-0)	0 (0-0)

Mean (standard deviation) or Median (first quartile, third quartile) values of synovial fluid parameters

Treatment	Day	Total protein (g/100 mL)	Total nucleated cell counts/µL	PGE2 (pg/mL)	CTX II (pg/mL)
Placebo	0	2 (0.2)	84,5 (40-116)	407 (367-429)	131 (86-192)
	7	2.1 (0.2)	141 (90-192)	386 (335-479)	145 (76-214)
	14	1.9 (0.2)	119 (70-160)	343 (289-433)	139 (126-236)
	28	1.7 (0.2)	98 (45-158)	406 (350-438)	148 (122-190)
Autologous BM-MSCs	0	2 (0.2)	62 (39-139)	401 (358-544)	136 (96-172)
	7	2.7 (1)	869 (143-2254)	432 (247-793)	106 (67-147)
	14	2.3 (0.5)	276 (140-1558)	444 (281-606)	102 (71-150)
	28	2 (0.7)	342 (81-459)	374 (198-432)	122 (115-141)
Allogeneic BM-MSCs	0	2(0.2)	71 (59-187)	459 (314-546)	132 (85-182)
	7	2.4 (0.6)	1110 (203-2805)	313 (210-426)	91 (62-182)
	14	2.1 (0.4)	2445 (128-3440)	401 (279-495)	92 (76-157)
	28	1.9 (0.3)	497 (176-687)	332 (213-426)	150 (103-192)
Allogeneic UCB-MSCs	0	2(0.2)	89 (47-130)	419 (343-510)	131 (91-162)
	7	2.5 (0.9)	614 (317-2268)	354 (235-418)	95 (53-128)
	14	2.3 (0.6)	864 (199-1739)	335 (265-386)	96 (82-131)
	28	2.2 (0.8)	479 (270-692)	360 (321-393)	146 (117-189)

Mean (standard deviation) or Median (first quartile, third quartile) values of synovial fluid parameters

Dosage	Day	Total protein (g/100 mL)	Total nucleated cell counts/µL	PGE2 (pg/mL)	CTX II (pg/mL)
10 million	0	2 (0.3)	91 (46-187)	421 (368-513)	85 (77-103)
	7	2.9 (1.1)	2095 (744-4090)	387 (325-435)	59 (35-87)
	14	2.2 (0.6)	1374 (361-2310)	404 (382-479)	88 (67-128)
	28	2.1 (0.7)	469 (436-644)	374 (274-393)	128 (118-145)
20 million	0	2 (0.1)	69 (46-124)	397 (261-560)	167 (139-265)
	7	2.2 (0.3)	236 (129-1955)	279 (207-565)	145 (210-187)
	14	2.2 (0.5)	204 (83-2547)	284 (201-418)	119 (84-189)
	28	2.0 (0.5)	261 (61-622)	339 (291-513)	150 (114-205)
