# Supplementary material for: Intra-Articular Injection of 2 Different Dosages of Autologous and Allogeneic Bone Marrow- and Umbilical Cord-Derived Mesenchymal Stem Cells Triggers a Variable Inflammatory Response of the Fetlock Joint on 12 Sound Experimental Horses
Source: Stem Cells Int. 2019 May 2;2019:9431894. doi: 10.1155/2019/9431894 (PMC6525957; doi:10.1155/2019/9431894)
Supplement: Supplementary 4 — Figure S1: synovial fluid sampling followed by intra-articular injection of MSCs or placebo in a metatarsophalangeal joint of one horse using a lateral approach on the flexed limb. [file 9431894.f4.pdf]

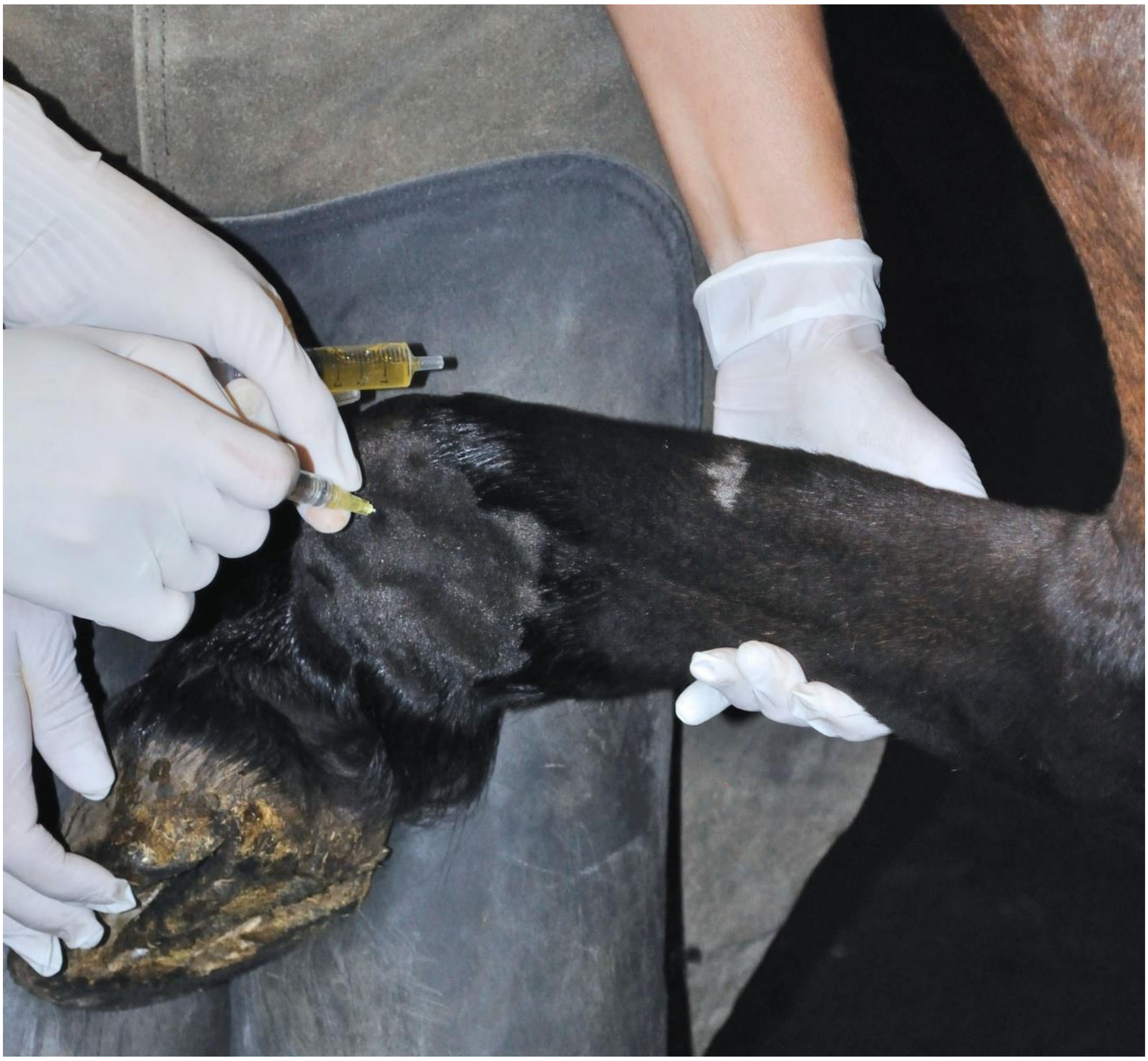

Figure S1: Synovial fluid sampling followed by intra-articular injection of MSCs or placebo in a metatarsophalangeal joint of one horse using a lateral approach on the flexed limb
