# Supplementary material for: Intra-Articular Injection of 2 Different Dosages of Autologous and Allogeneic Bone Marrow- and Umbilical Cord-Derived Mesenchymal Stem Cells Triggers a Variable Inflammatory Response of the Fetlock Joint on 12 Sound Experimental Horses
Source: Stem Cells Int. 2019 May 2;2019:9431894. doi: 10.1155/2019/9431894 (PMC6525957; doi:10.1155/2019/9431894)
Supplement: Supplementary 5 — Figure S2: ultrasound technique and reference images. [file 9431894.f5.pdf]

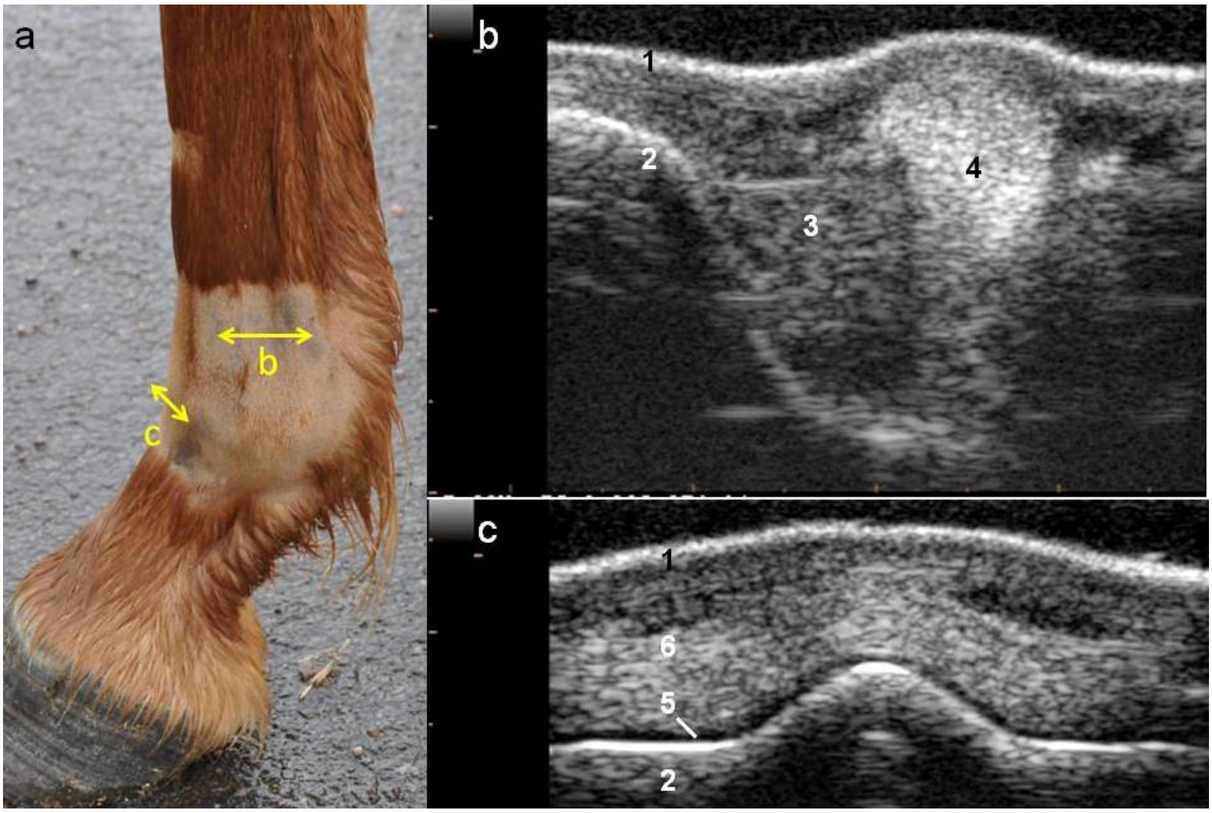


**Figure S2:** Ultrasound technique and reference images. (a) Latéro-médial photograph of the left fore fetlock of horse 1 showing the position of the probe used to perform ultrasound images (b) and (c) (yellow double arrows); (b) Transverse ultrasound scan of the lateral aspect of the fetlock, dorsal is to the left. Note the concave aspect of the skin at the level of the synovial recess on reference image; (c) Transverse ultrasound scan of the dorsal aspect of the fetlock, medial is to the left. Note the absence of synovial fluid effusion on reference image. 1- Skin; 2- Third metacarpal bone; 3- proximo-palmar recess of the metacarpo-phalangeal joint; 4- Lateral branch of the suspensory ligament (third interosseous muscle); 5- Articular cartilage of the metacarpal condyle; 6- Capsula.
