# Supplementary material for: Intra-Articular Injection of 2 Different Dosages of Autologous and Allogeneic Bone Marrow- and Umbilical Cord-Derived Mesenchymal Stem Cells Triggers a Variable Inflammatory Response of the Fetlock Joint on 12 Sound Experimental Horses
Source: Stem Cells Int. 2019 May 2;2019:9431894. doi: 10.1155/2019/9431894 (PMC6525957; doi:10.1155/2019/9431894)
Supplement: Supplementary 6 — Figure S3: characterization of equine bone marrow MSCs and umbilical cord blood MSCs. [file 9431894.f6.pdf]

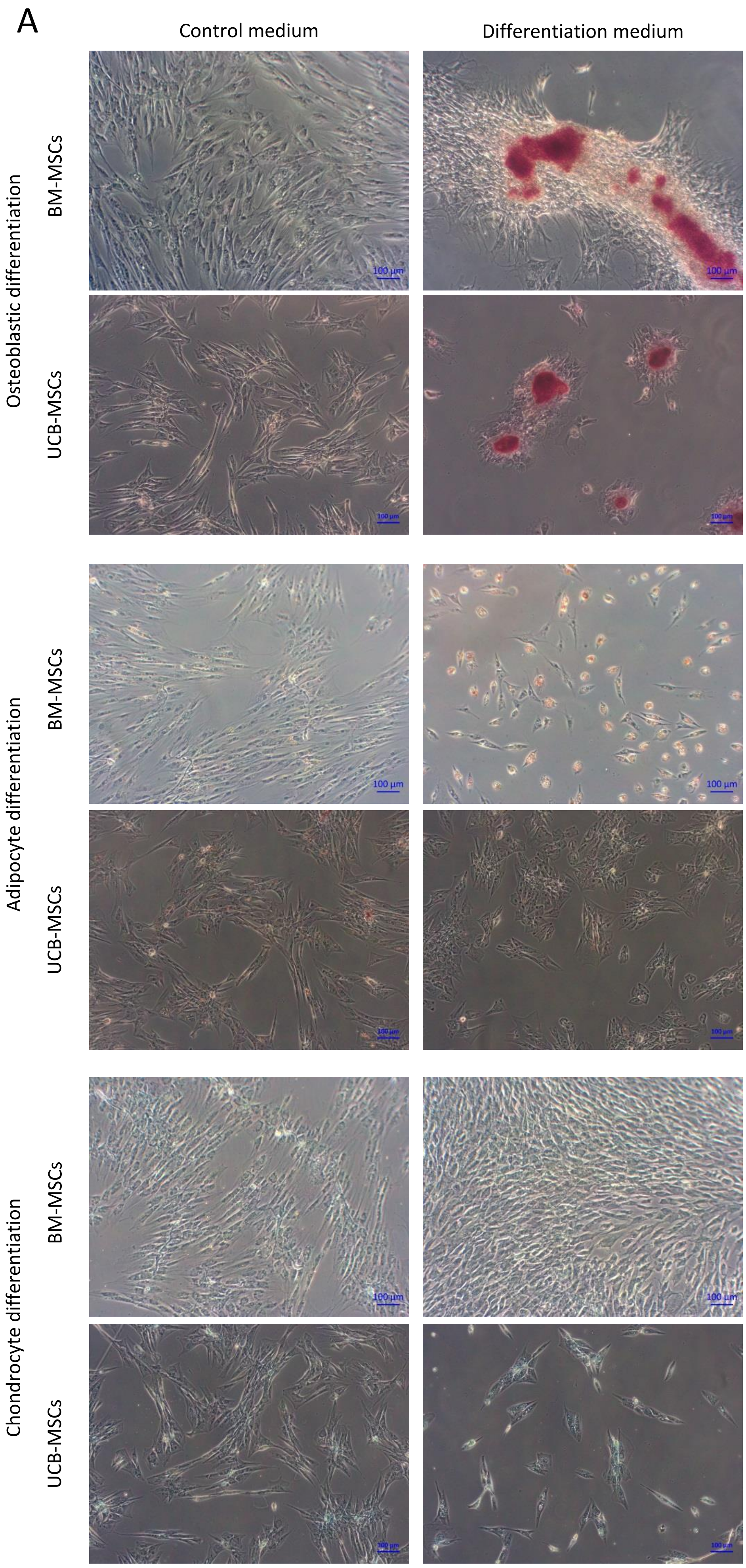

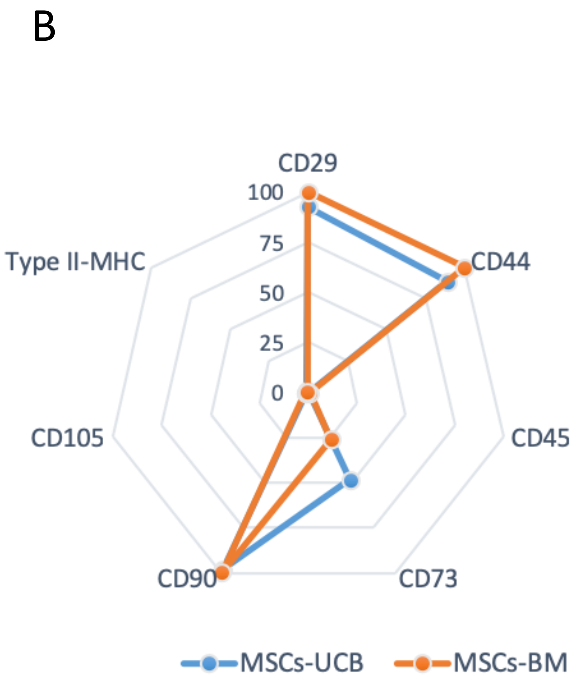

Figure S3 : Characterization of equine bone marrow (BM)-MSCs and umbilical cord blood (UCB)-MSCs.

(A) Multipotencyability of MSCs to differentiate into osteoblasts, adipocytes and chondrocytes. The cells were amplified at P4 and cultured at 50% confluence with media specific for osteoblastic, adipocyte or chondrogenic differentiation (right panel) or amplification medium (left panel) for 14 days. After fixing with paraformaldehyde, cells were stained with Alizarin red, Oil Red O and Alcian blue to respectively reveal the presence of calcium deposits, lipid droplets and sulfated proteoglycans. The results present one representative strain.

(B) Immunophenotyping of the isolated and amplified MSCs. The cells were detached and suspended at 10 million cells per ml in PBS. They were incubated with the indicated specific monoclonal antibodies, or relevant anti-IgG isotype controls for 30 min at 4 °C in a dark room. The cells were then washed and resuspended in PBS. The acquisitions involved a minimum of 10,000 events collected for each analysis. The results are expressed as the mean of the values obtained for 4 strains of BM-MSCs and 4 strains of UCB-MSCs. CD: cluster of differentiation ; MHC: major histocompatibility complex
