# Supplementary material for: Intra-Articular Injection of 2 Different Dosages of Autologous and Allogeneic Bone Marrow- and Umbilical Cord-Derived Mesenchymal Stem Cells Triggers a Variable Inflammatory Response of the Fetlock Joint on 12 Sound Experimental Horses
Source: Stem Cells Int. 2019 May 2;2019:9431894. doi: 10.1155/2019/9431894 (PMC6525957; doi:10.1155/2019/9431894)
Supplement: Supplementary 7 — Table S1: distribution of MSC treatments. Table S2: quality control of MSCs. Table S3: sensitivity to digital flexion tests grading system. Table S4: subcutaneous oedema grading system. [file 9431894.f7.pdf]

Supplementary Tables

Table S1: Distribution of stem cell treatments

	LF	RF	LH	RH
Distribution 1 (horses 1 and 7)	Allogenic BM-MSCs	Autologous BM-MSCs	PBS	Allogenic UCB-MSCs
Distribution 2 (horses 2 and 8)	Allogenic BM-MSCs	Autologous BM-MSCs	Allogenic UCB-MSCs	PBS
Distribution 3 (horses 3 and 9)	Autologous BM-MSCs	Allogenic BM-MSCs	PBS	Allogenic UCB-MSCs
Distribution 4 (horses 4 and 10)	Allogenic UCB-MSCs	PBS	Autologous BM-MSCs	Allogenic BM-MSCs
Distribution 5 (horses 5 and 11)	Allogenic UCB-MSCs	PBS	Allogenic BM-MSCs	Autologous BM-MSCs
Distribution 6 (horses 6 and 12)	PBS	Allogenic UCB-MSCs	Autologous BM-MSCs	Allogenic BM-MSCs

LF – Left front fetlock ; RF – Right front fetlock ; LH– Left hind fetlock ; RH – Right hind fetlock
MSCs – Mesenchymal stem cells; PBS – Phosphate Buffered Saline; BM – Bone marrow; UCB – Umbilical cord blood

Table S2: Quality control of MSCs – List of pathogens

Equine viral arteritis
Equine influenza virus
Equine coronavirus
Type A equine rhinovirus
Type B equine rhinovirus
Type 1 equine adenovirus
Type 2 equine adenovirus
Equine hepacivirus
Herpesviruus
Aerobic and anaerobic bacteria
Yeasts, molds and aerobic bacteria
Lyme disease (*Borrelia*)
Mycoplasma
Q fever (*Coxiella burnetti*)
Ehrlichiosis (*Anaplasma phagocytophilum*)
Pathogenic Leptospira
Chlamydia infection (*Chlamydophila abortus, Chlamydophila psittaci*)
Piroplasmosis (*Babesia caballi*)
Piroplasmosis (*Theileria equi*)

Table S3: Sensitivity to digital flexion tests grading system

Score		Physical criteria
0	Normal	No reaction of the horse to the flexion of the fetlock joint when using a moderate strength (3kg).
1	Mild	Reaction of the horse (withdrawal of the limb) to the flexion of the fetlock joint when using a moderate strength (3kg).
2	Moderate	Reaction of the horse (withdrawal of the limb) to the flexion of the fetlock joint when using a light strength (1kg).
3	Substantial	Reaction of the horse (withdrawal of the limb) to the flexion of the fetlock joint before using strength.
4	Severe	Violent withdrawal of the limb when flexion the fetlock joint without stregth

Table S4: Subcutaneous oedema grading system

Score		Physical criteria
0	Normal	No oedema
1	Mild	Minimal oedema at the injection site
2	Moderate	Mild oedema over the fetlock joint
3	Substantial	Oedema extending distally to the pastern and / or proximally to the metacarpus
4	Severe	Oedema extending proximally to the carpus or hock
